# Science-based decision support for formulating crop fertilizer recommendations in sub-Saharan Africa

**DOI:** 10.1016/j.agsy.2020.102790

**Published:** 2020-04

**Authors:** Jairos Rurinda, Shamie Zingore, Jibrin M. Jibrin, Tesfaye Balemi, Kenneth Masuki, Jens A. Andersson, Mirasol F. Pampolino, Ibrahim Mohammed, James Mutegi, Alpha Y. Kamara, Bernard Vanlauwe, Peter Q. Craufurd

**Affiliations:** a International Plant Nutrition Institute (IPNI), c/o IFDC – East & Southern Africa Division, ICIPE Compound, Duduville – Kasarani, Thika Road, P. O. Box 30772-00100, Nairobi, Kenya; b African Plant Nutrition Institute, Lot 666 Hay, Moulay Rachid, 43150, Benguerir, Morocco.; c Centre for Dryland Agriculture (CDA), Bayero University Kano, 70001 Kano, Nigeria; d Ethiopian Institute of Agricultural Research, P.O.Box 2003, Addis Ababa, Ethiopia; e International Maize and Wheat Improvement Center (CIMMYT), Selian Agricultural Research Institute, CIAT Building, Off Dodoma Road, P. O. Box 2704, Arusha, Tanzania; f International Maize and Wheat Improvement Center (CIMMYT)- Southern Africa, c/o Knowledge, Technology and Innovation Group, Wageningen University, P.O. Box 8130, 6700, EW, Wageningen, the Netherlands; g International Plant Nutrition Institute, c/o Crop and Environmental Sciences Division, International Rice Research Institute (IRRI), DAPO Box 7777, Metro Manila 1301, Philippines; h International Institute of Tropical Agriculture, PMB 5320, Oyo Road, Ibadan 200001, Oyo State, Nigeria; i International Institute of Tropical Agriculture (IITA), c/o ICIPE Compound, P. O. Box 30772-00100, Nairobi, Kenya; j CIMMYT South Asia Regional Office, NARC Research Station, Khumaltar, Lalitpur, Kathmandu, Nepal

**Keywords:** Fertilizer recommendations, Nutrient use efficiency, Yield response, Maize intensification, Soil fertility management, Smallholder farming systems

## Abstract

In sub-Saharan Africa, there is considerable spatial and temporal variability in relations between nutrient application and crop yield, due to varying inherent soil nutrients supply, soil moisture, crop management and germplasm. This variability affects fertilizer use efficiency and crop productivity. Therefore, development of decision systems that support formulation and delivery of site-specific fertilizer recommendations is important for increased crop yield and environmental protection. Nutrient Expert (NE) is a computer-based decision support system, which enables extension advisers to generate field- or area-specific fertilizer recommendations based on yield response to fertilizer and nutrient use efficiency. We calibrated NE for major maize agroecological zones in Nigeria, Ethiopia and Tanzania, with data generated from 735 on-farm nutrient omission trials conducted between 2015 and 2017. Between 2016 and 2018, 368 NE performance trials were conducted across the three countries in which recommendations generated with NE were evaluated relative to soil-test based recommendations, the current blanket fertilizer recommendations and a control with no fertilizer applied. Although maize yield response to fertilizer differed with geographic location; on average, maize yield response to nitrogen (N), phosphorus (P) and potassium (K) were respectively 2.4, 1.6 and 0.2 t ha^−1^ in Nigeria, 2.3, 0.9 and 0.2 t ha^−1^ in Ethiopia, and 1.5, 0.8 and 0.2 t ha^−1^ in Tanzania. Secondary and micronutrients increased maize yield only in specific areas in each country. Agronomic use efficiencies of N were 18, 22 and 13 kg grain kg^−1^ N, on average, in Nigeria, Ethiopia and Tanzania, respectively. In Nigeria, NE recommended lower amounts of P by 9 and 11 kg ha^−1^ and K by 24 and 38 kg ha^−1^ than soil-test based and regional fertilizer recommendations, respectively. Yet maize yield (4 t ha^−1^) was similar among the three methods. Agronomic use efficiencies of P and K (300 and 250 kg kg^−1^, respectively) were higher with NE than with the blanket recommendation (150 and 70 kg kg^−1^). In Ethiopia, NE and soil-test based respectively recommended lower amounts of P by 8 and 19 kg ha^−1^ than the blanket recommendations, but maize yield (6 t ha^−1^) was similar among the three methods. Overall, fertilizer recommendations generated with NE maintained high maize yield, but at a lower fertilizer input cost than conventional methods. NE was effective as a simple and cost-effective decision support tool for fine-tuning fertilizer recommendations to farm-specific conditions and offers an alternative to soil testing, which is hardly available to most smallholder farmers.

## Highlights

• A nutrient management tool balanced fertilizer P & K, and increased farm profits
• Maize yield response to fertilizer differed widely with geographic location
• Plant secondary and micronutrients increased maize yield only in specific areas
• Agronomic use efficiency of fertilizer is low in African smallholder systems
• Use of nutrient management tools can be an alternative to soil test-based method

## 1 Introduction

The formulation of fertilizer recommendations tailored to specific crops, climate and soil fertility conditions, as well as farmers' socio-economic status can increase productivity, and reduce climate-related production risks and undesirable impacts of fertilizer on the environment. The need for such specific recommendations is much more so in diverse farm types, in different soil and climatic conditions (Mtambanengwe and Mapfumo, 2005; Zingore et al., 2007; Giller et al., 2011), and in crop systems most vulnerable to changing rainfall patterns (Rurinda et al., 2015; Nezomba et al., 2018), such as in sub-Saharan Africa (SSA). However, the irony is that agricultural advisory services in SSA have been promoting blanket fertilizer recommendations in which a single fertilizer rate is used for large but heterogeneous areas. This leads to unbalanced application of fertilizer nutrients relative to the needs of a crop, and low use efficiency of fertilizer. Further, despite their promotion, many smallholder farmers hardly afford the recommended quantities of fertilizers. Current fertilizer nutrient application rates in SSA average only about 16 kg ha^−1^ year^−1^, compared with over 100 kg ha^−1^ in Europe and North America, and over 150 kg ha^−1^ in China in Asia (IFASTAT, 2019). Blanket fertilizer recommendations have been developed with a conventional ‘top-down’ method, which uses a limited number of field-experimental data points, as investment requirements to conduct such fertilizer trials are considerable. With evolution of computer-based modelling and decision support systems (which have capabilities to simplify and solve complex systems problems by integrating empirical and farmers' knowledge) reliable and cost effective fertilizer guidelines can be developed and delivered quickly. Given that there is increasing number of fertilizer companies that produce more site- and crop-specific fertilizer types in SSA, these decision support systems can also leverage efforts from fertilizer companies to support better access for farmers to knowledge of their soils and crops to adapt fertilizer and integrate technologies to optimize yield.

Crop production models have been developed and widely used to test hypotheses, run virtual experiments, and perform scenario and risk analyses at different scales, and enhanced the scientific understanding of complex interactions between soil, crop, environment and management. Examples of such models are WOFOST (van Diepen et al., 1989), DSSAT (Jones, 1993) and APSIM (Keating et al., 2003). Because of their complexity and high demand for input data (i.e. data for model development, evaluation and use) that are seldom available for much of SSA, these models have hardly been used to package and deliver scientific knowledge in a way that can be used by policy makers, extension advisers and farmers. Studies have shown that policy makers, agricultural extension advisers and farmers can only make use of decision support tools when they perform well, are simple, cost-effective and relevant to the user (Rose et al., 2016). The model QUEFTS (Quantitative Evaluation of Soil Fertility of Tropical Soils), which accounts for interactions among macro-nutrients to estimate balanced nutrient requirements for a crop target yield at a specific location, is generic and requires limited input data (Janssen et al., 1990). It has been calibrated and validated for different crops in varying soils, climate and management conditions in sub-Saharan Africa (Smaling and Janssen, 1993; Haefele et al., 2003; Ezui et al., 2016) and other regions (Witt et al., 1999; Sattari et al., 2014). Consequently, the QUEFTS model enables the development of simple and cost-effective decision support tools for nutrient management and fertilizer recommendations. One such decision support tool is Nutrient Expert (Pampolino et al., 2012).

Nutrient Expert (NE) is a simple, computer or mobile phone based decision support tool, developed with a method based on QUEFTS and on-site agronomic information (i.e. climate, inherent soil fertility conditions of the targeted field, previous crop and nutrient management, current and expected yield, availability of fertilizer types, farm input-output prices and farmer objectives) (Pampolino et al., 2012). It provides a systematic method to develop strategies for balanced nitrogen (N), phosphorus (P), potassium (K), secondary- and micro-nutrients use on a specific field or in a larger area with similar growing conditions. NE is designed for agricultural extension advisers to provide advice to farmers on best crop management practices and to help farmers maximize the benefits of their investment in fertilizers. It has been developed in a participatory manner, involving researchers, extension service providers and farmers to address their needs.

The agronomic and economic performance of NE recommendations has been comprehensively evaluated in Asia (Xu et al., 2014, Xu et al., 2019), where NE use increased maize yield by between 0.9 and 1.6 t ha^−1^ and profits by between US$ 270 and US$ 380 ha^−1^ as compared to farmers' fertilizer practices (Pampolino et al., 2012). In much more variable and complex farming systems in SSA, such evaluations have been limited. Further, in order to convince agriculture planners and extension advisers of the value of nutrient management decision support tools such as NE, it is crucial to initially evaluate them relative to prevailing blanket recommendations and commonly accepted methods such as soil testing. Although widely used, soil testing method has several challenges that limit its use in smallholder farming systems in SSA, such as: high costs of soil sampling and analysis, the difficulty in taking representative soil samples, ill-equipped laboratories and the time required to produce results. There are also limitations associated with interpretation of soil test results (Njoroge et al., 2017).

The main objective of this study was to evaluate soil nutrient constraints for maize production in major maize-based farming systems in Nigeria, Ethiopia and Tanzania, and to use this for the calibration and validation of NE. The study specifically sought to: (i) estimate maize yield response to nutrients supplied from fertilizers and calculate agronomic use efficiencies of N, P and K, (ii) calibrate NE for maize for a wide range of cropping systems, soil and climatic conditions, and (iii) to gain insight into the agronomic and economic benefits of NE recommendations relative to soil-test based and blanket fertilizer recommendations. The focus was on maize (*Zea mays* L.), as this is a strategic staple crop for achieving food security in SSA. The study was conducted in smallholder farming systems in Nigeria, Ethiopia and Tanzania as together, these countries comprise one-third of the human population of SSA.

## 2 Materials and methods

### 2.1 Study area selection and description

**Fig. 1:**
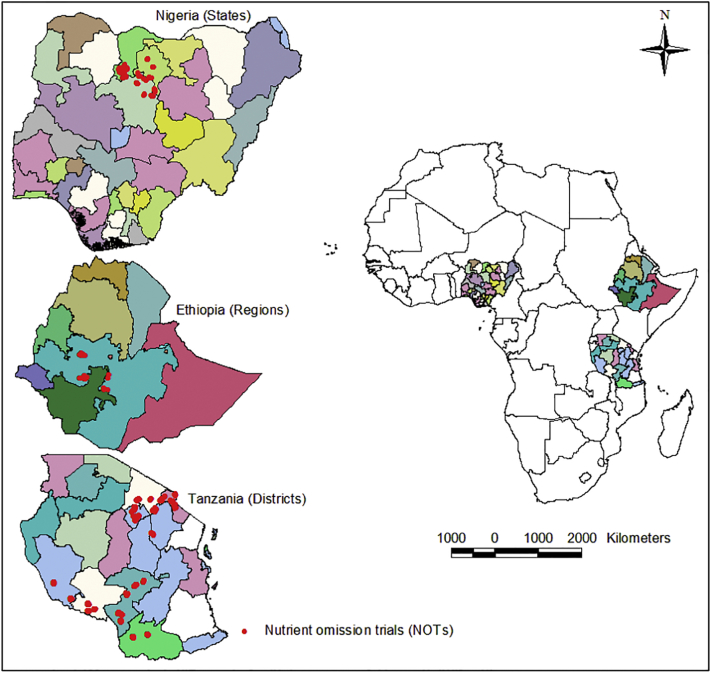
Geo-spatial distribution of nutrient omission trials (NOTs) in the studied areas in Nigeria, Ethiopia and Tanzania.

We calibrated and evaluated Nutrient Expert (NE) for major maize agroecological zones in Nigeria, Ethiopia and Tanzania between 2015 and 2018. In each country, the study areas were selected based on three main criteria: (i) large coverage of major maize producing areas (classified with the Africa Soil Information Service - AfSIS) scheme; (ii) areas with research and development programs that can support the scaling of nutrient management decision support tools, (iii) areas with relatively high human population densities (i.e. >25 persons km^2^) and good market access (within 3 h of an urban market), for intensification of maize production. Consequently, the study was conducted in three states of Nigeria: Kaduna, with testing sites in Giwa, Ikara, Kauru, lere, Makarfi and Soba local government areas (LGAs); Kano, with testing sites in Bunkure, Doguwa, Tofa and Tudun Wada LGAs; and Katsina, with testing sites in Bakori, Dandume, Faskari and Funtua LGAs (Fig. 1) (Shehu et al., 2018). In Ethiopia, the study was conducted in the Jimma, Bako, Hawassa, Bulbula and Adami Tullu areas (Balemi et al., 2019). In Tanzania it was conducted in the southern highlands, with testing sites in Iringa Rural, Kilolo, Ludewa, Mbeya Rural, Mbozi, Mufindi, Namtumbo, Njombe Rural, Nkasi, Songea Rural and Sumbawanga Rural districts; and in northern zone with testing sites in Arumeru, Babati Rural, Hai, Hanang, Karatu, Kiteto, Mbulu, Monduli, Moshi Rural, Mwanga and Rombo districts (Fig. 1). The study areas covered a wide range of climatic and soil conditions, cropping systems and farm types, and socio-economic conditions (Table 1). We characterized a few soil properties (i.e. soil organic carbon (SOC), P and texture, Table 1), which are relatively stable in the soil and hence assumed to be good indicators of soil fertility in the context of African smallholder farming systems. In Nigeria, the study areas are characterized by unimodal rainfall from May to November. In Adami Tullu and Hawassa in Ethiopia, the rainfall is bimodal with the short rains from March to May and the long rains from June to November. In Jimma and Bako areas in Ethiopia, the rainfall is unimodal from May to November. The rainfall in the northern zone of Tanzania is bimodal with the short rains from March to June, and the long rains from October to December. In the southern highlands of Tanzania the rainfall is unimodal from November to May.

**Table 1:** Characteristics of the study areas in Nigeria, Ethiopia and Tanzania; the values are means with minimum and maximum (range) in brackets.

Country	Study area	Soil properties					Rainfall	Temperature	Major crops	Common fertilizer	farm size	Farmer maize yield
		pH (H_2_O)	SOC (g kg^−1^)	Av. P (mg kg^−1^)	Clay (%)	Sand (%)	mm	°C			ha	t ha^−1^
Nigeria	Kaduna	5.7(5.0–6.4)	8(3−13)	5.3(1.6–20.7)	22(13–36)	48(32–70)	1217	25.8	Maize, cowpea, soybean, sorghum & rice	NPK, Urea	1.6(0.2–5.0)	2.4(0.0–9.0)
	Kano	6.2(5.3–7.2)	8(3–15)	4.6(1.4–11.3)	23(12–42)	53(26–76)	1150	26.9	Maize,soybean, sorghum, rice & millet	NPK, Urea	1.8(0.5–3.0)	2.1(0.0–6.5)
	Katsina	5.9(5.1–6.6)	8(5–12)	4.4(1.9–12.7)	21(14–32)	21(14–32)	582	26.9	Maize, sorghum & soybean	NPK, Urea	1.4(0.2–4.0)	1.9(0.0–9.0)
Ethiopia	Jimma / Bako	5.1(4.6–5.8)	22(13–29)	9.0(3.9–61.8)	59(36–78)	16(6–38)	1215	19.3	Maize, teff, sorghum & pepper	NPS, Urea	1.7(0.1–10.0)	4.0(0.6–7.0)
	Adami Tullu / Bulbula	7.2(6.7–7.9)	8(6–11)	34(11.9–61.4)	–	–	623	19.4	Maize, haricot, common bean & sorghum	NPS, Urea	2(0.2–5.0)	3.5(0.9–7.3)
	Hawassa	7.1(6.7–7.4)	6(4–7)	31(18.2–55.7)	–	–	602	19.9	Maize, haricot, common bean & sorghum	NPS, Urea	0.9(0.4–1.8)	3.7(1.2–8.7)
Tanzania	Southern highlands	6.0(4.7–7.0)	15(3–70)	8.5(0.1–54.5)	25(9–54)	60(32–85)	928	22.8	Maize, common bean & sunflower	DAP,Urea	5.0(1.0–10.0)	2.6(0.1–10.9)
	Northern highlands	6.8(4.7–8.6)	15(2–58)	11(0.4–33.5)	27(16–60)	58(20–74)	874	17.3	Maize, banana & common bean	DAP, Urea	4.2(1.2–8.1)	2.2(0.1–10.0)

pH – soil pH in water (soil/water of 1:1); SOC - soil organic carbon (a modified Walkley & Black chromic acid wet chemical oxidation & spectrophotometric method); Av. P – Available phosphorus (Mehlich- 3 extraction procedure); texture (hydrometer method). Rainfall – long-term mean annual total; Temperature – long-term average; N – nitrogen; P – phosphorus; K – potassium; S – sulfur; DAP – diammonium phosphate. Soil data were analysed in this study (texture data for Adami Tullu and Hawassa areas were not analysed); and farm characterization data were summarized from a survey data drawn from the project: Taking Maize Agronomy to Scale in Africa (TAMASA) (https://tamasa.cimmyt.org).

### 2.2 Nutrient Expert development and calibration

#### 2.2.1 The nutrient expert decision support tool

Nutrient Expert is based on the QUEFTS model (Janssen et al., 1990), and follows the principles and guidelines of site-specific nutrient management (SSNM) (Dobermann and White, 1998; Pampolino et al., 2012). The aims of SSNM are to (i) supply a crop's total nutrient uptake requirements tailored to a specific field or larger area with similar growing conditions, (ii) account for nutrients supplied by the soil including nutrients from organic sources such as crop residue and manure, (iii) apply fertilizer at optimal rates and at critical growth stages to bridge the deficit between the nutrient needs of a crop and the nutrients supplied from the soil; and (iv) account for net P and K offtake in harvested produce to maintain long-term soil fertility status for sustainable crop production. NE integrates all this information to determine crop nutrient input requirements. Each of N, P and K input requirement (kg nutrient ha^−1^) of a crop is calculated with an Eq. (1):

(1) $$N_{i}=\left(U_{i}–S_{i}\right)/{\mathrm{RE}}_{i}$$

where N is the crop nutrient input requirement; *i* is N, P or K; U is the whole-crop nutrient uptake for attainable yield; S is the amount of a nutrient supplied from the soil; and RE is the maximum recovery fraction of the applied nutrient (*i*).

The whole-crop nutrient uptake (U_*i*_) is calculated from the attainable yield (Y_a_) and internal nutrient use efficiency (IE, i.e. the relation between grain yield and balanced uptake of nutrients at harvest in kg grain kg^−1^ nutrient in above ground plant dry matter) with an eq. (2):

(2) $$U_{i}=Y_{a}/{\mathrm{IE}}_{i}$$

The attainable yield (Y_a_) is the yield of a crop for a typical growing season at a location using best management practices without nutrient limitation. It is determined from the NPK treatment in nutrient omission trials (NOTs) if no other deficiencies are yield-limiting. These trials aim to establish crop responses to N, P and K (and in this study also a specific combination of micro-nutrients); described subsequently through treatments that omit at least one nutrient of interest while applying other in ample amounts to determine the limiting effect of the nutrient of interest. The IE is predicted from the QUEFTS envelope functions (Janssen et al., 1990). The QUEFTS model requires the estimation of the minimum and maximum internal concentrations of the macronutrients N, P and K in the economic product of a crop and its residues (i.e. the estimation of the upper and lower borderlines describing the minimum and maximum internal efficiencies). In this study the borderlines excluded 2.5% of upper and lower extreme values and observations with harvest index (HI) of <0.4 as the data with low HI was assumed to be from a crop suffering from water, poor soil fertility, biotic or abiotic stress (Xu et al., 2019). The NE uses IE for a linear function until a relative yield of about 70–80% of the genetic maize yield potential.

The RE, which is the ratio of crop nutrient uptake to nutrients applied from fertilizer, is calculated with an Eq. (3):

(3) $${\mathrm{RE}}_{i}=\left(\text{Uptake of}\;a\;\text{nutrient}\;\left(i\right)\;\text{from an}\;\mathrm{NPK}\;\text{plot}–\text{Uptake of}\;a\;\text{nutrient}\;\left(i\right)\;\text{from nutrient}\;\left(i\right)\;\text{limited plot}\right)/\text{Total nutrient}\;\left(i\right)\;\text{applied from fertilizer}$$

The QUEFTS envelope functions and recovery fractions of N, P and K were calibrated for each study area in each country with fertilizer applications, grain yield and nutrient uptake datasets generated from the NOTs.

The amount of a nutrient (*i*) supplied from the soil (S) is estimated from the nutrient-limited yield, which is the yield achieved when only the nutrient (*i*) of interest is omitted (is determined from the NOTs). The indigenous N soil supply determines the ratio of N-limited yield (Y_s_) to attainable yield (Y_a_). Similarly, the indigenous P soil supply and indigenous K soil supply determine the ratio of the P-limited yield and K-limited yield, respectively, to Y_a_. The indigenous nutrient soil supply varies widely in space and time due to inherent soil properties, climate and agronomic farm management. To account for this variability, NE uses datasets from on-farm nutrient omission trials (NOTs) conducted in a wide range of soil, climate and farm management conditions. Then the 25th percentile, median, and 75th percentile of all nutrient omission trials data for the ratio of Y_s_ to Y_a_ are used as coefficients to estimate the nutrient limited yield for a given attainable yield and soil fertility class. The median represents soils with ‘average’ fertility, and the 25th and 75th percentile represent ‘low’ and ‘high’ fertility, respectively.

Given that the amount of nutrients taken up by a crop is directly related to its yield, the attainable yield indicates the total nutrient requirement and the nutrient-limited yield indicates the indigenous soil nutrient supply (Pampolino et al., 2012). Therefore, the crop's nutrient input requirements can be estimated from the expected yield response to each fertilizer nutrient and agronomic fertilizer use efficiency. Therefore, the eq. (1) mentioned above is similar to an eq. (4), which is used in NE:

(4) $$N_{i}=\left(Y_{a}–Y_{s}\right)/\mathrm{AE}$$

where N is the crop nutrient input requirement; Y_a_ is attainable yield; Y_s_ is the nutrient limited yield; and AE is the agronomic efficiency of applied fertilizer nutrient input.

The yield response is the difference between the Y_a_ and Y_s_. In other words, the yield response to N, P or K is the yield gap between NPK plots that received ample nutrients and omission plots when one of the nutrients is omitted. The yield response indicates the nutrient deficit, which must be supplied by fertilizers. The AE is kg extra grain per kg nutrient applied and was calculated with data from NOTs with an eq. (5).

(5) $${\mathrm{AE}}_{i}=\left(\text{Yield from an}\;\mathrm{NPK}\;\text{plot}–\text{Yield from nutrient}\;\left(i\right)\;\text{limited plot}\right)/\text{Total nutrient}\;\left(i\right)\;\text{applied from fertilizer}$$

Overall in NE, the crop N input requirements are determined based on yield response to fertilizer and agronomic efficiencies of N (the eq. 4). The determination of fertilizer P and K requirements has been modified to consider the internal nutrient efficiency, attainable yield, nutrient balances, yield responses and residual nutrients from the previous crop (Pampolino et al., 2012; Xu et al., 2019) and calculated with eqs. (6 and 7).

(6) $$\text{Crop}\;P\;\text{input requirement}={\left(Y_{a}–Y_{s}\right)}_{P}\times {\mathrm{RIE}}_{P}/{\mathrm{RE}}_{P}+Y_{a}\times {\mathrm{RIE}}_{P}\times {\mathrm{HI}}_{P}\times X_{G}\%)$$

(7) $$\text{Crop}\;K\;\text{input requirement}={\left(Y_{a}–Y_{s}\right)}_{K}\times {\mathrm{RIE}}_{K}/{\mathrm{RE}}_{K}+Y_{a}\times \left({\mathrm{RIE}}_{K}\times {\mathrm{HI}}_{K}\times 100\%+\mathrm{RIE}\times \left(1-{\mathrm{HI}}_{K}\right)\times X_{S}\%\right)$$

where (Y_a_ – Y_s_)_i_ is yield response (kg ha^−1^), RIE is nutrient uptake requirement per ton of grain yield (kg ha^−1^), RE is recovery efficiency to nutrient application (%), Ya is attainable yield (kg ha^−1^), HI is harvest index, X_G_% and X_S_% are the nutrient return proportion of grain and straw, respectively.

Further information on the development and parameterization of NE can also be found in the selection from Pampolino et al. (2012).

#### 2.2.2 Nutrient omission trials (NOTs)

Nutrient omission trials (NOTs) with maize were conducted in farmers' fields in the studied areas in Nigeria (*N* = 423), Ethiopia (*N* = 148), and Tanzania (*N* = 300), following a standardized experimental protocol. The trials were conducted over two agricultural seasons in Nigeria and Ethiopia in 2015 and 2016, and over one season (2016–2017) in Tanzania. In Tanzania, the trials were conducted for only one season and this was assumed to suffice as the trials were conducted across a wide range of soil, climate and farm management conditions. In Adami Tullu and Hawassa in Ethiopia, and in the northern zone of Tanzania, the trials were conducted during the long rainy seasons only. Experimental fields were selected by delineating each study area into 10 × 10 km grids with ArcGIS software. Each of these 10 × 10 km grids was further delineated into 1 × 1 km sub-grids. A total of five 1 × 1 km sub-grids were selected in each study area to represent major climatic conditions, soil type, common cropping systems and farm management conditions with the aid of agro-ecological maps and local researchers. In each selected sub-grid, a field for experimentation was randomly selected, taking into account the willingness of a farmer to host the trial and availability of land to accommodate all six treatments (described subsequently).

The trials comprised six treatments: (i) a control, (ii) an NPK, (iii) N omitted from NPK, (iv) P omitted from NPK, (v) K omitted from NPK, and (vi) secondary and micronutrients added to NPK. The trials were laid out in a randomized complete design replicated across farm. Plot sizes of 8 × 8 m were used in Ethiopia and Tanzania. In Nigeria, a plot size of 5 × 6 m was used because suitable fields were limited; and two trials had to be established side by side on each field – one to evaluate hybrid maize and the other one to evaluate an OPV.

The nutrients (N, P & K) were applied with straight fertilizers at rates estimated to achieve the expected attainable yield without nutrient limitation in each study area. The nutrients application rates were calculated depending on the maximum attainable yield as determined based on rainfall and agro-ecological potential (Table 2). Secondary and micronutrients were applied at 24 kg S ha^−1^ as sulphates of Ca, Mg and Zn, 10 kg Ca ha^−1^ as CaSO_4_, 10 kg Mg ha^−1^ as MgSO_4_, 5 kg Zn ha^−1^ as ZnSO_4_ and 5 kg B ha^−1^ as borax. Nitrogen was applied in three equal splits, i.e. at planting (basal), and at 21 and 35 days after emergence (DAE). All other nutrients were applied as basal at time of planting. Maize was planted at a spacing of 75 × 25 cm (1 plant per hole after thinning) equal to a plant population of 53,000 plants per hectare. A high yielding maize hybrid variety recommended for each study area was used as the test crop. In the Jimma and Bako areas of Ethiopia, the hybrid BH661 (with 160 average days to maturity) variety was used, while in the Hawassa and Adami Tullu areas BH540 (with 145 average days to maturity) was used. In Nigeria, the hybrid varieties used were OBA SUPER-9 (with 105–110 days to maturity) and OBA SUPER-1 (with 105–118 days to maturity) in the 2015 and 2016 seasons, respectively; and the OPV varieties used were IWD C2 SYN F2 (with 105–110 days to maturity) and EVDT W STR (with 90–95 days to maturity) in the northern Guinea savanna and Sudan savanna study sites, respectively. In Tanzania, a number of hybrid maize varieties were used, including SC 403 (with 131 days to maturity), SC 513 (with 137 days to maturity), SC 627 (with 142 days to maturity) each suitable to the locations in which they were planted.

**Table 2:** Nitrogen (N), phosphorous (applied as P_2_O_5_) and potassium (applied as K_2_O) application rates used in nutrient omission trials (NOTs) conducted in multiple locations in Nigeria, Ethiopia, and Tanzania.

Country	Study area	*Site growing condition	N kg ha^−1^	P kg ha^−1^	K kg ha^−1^
Nigeria	Kaduna	Most favourable	140	50	50
	Kano	Most favourable	140	50	50
		More favourable	120	40	40
	Katsina	Most favourable	140	50	50
Ethiopia	Jimma / Bako	More favourable	120	40	40
	Adami Tullu/bulbula	More favourable	120	40	40
	Hawassa	More favourable	120	40	40
Tanzania	Southern highlands & northern zone	Most favourable	140	50	50
		More favourable	120	40	40
		Favourable	100	30	30

*Most favourable: high rainfall and high potential maize production with attainable yield of 8–10 t ha^−1^; more favourable: moderate rainfall and medium potential maize production with attainable yield of 7–8 t ha^−1^; favourable: low rainfall and low potential maize production with attainable yield of 5–6 t ha^−1^.

The experimental fields were cleared and the residues from previous season's crops removed before ploughing and harrowing to a depth of 20 cm. The plots were weeded manually at least twice during each cropping season. Pests and diseases were monitored regularly and remedial action taken as required. The trials were managed by researchers, but with support from extension advisers and host farmers.

Maize grain yield was determined at physiological maturity from a net plot size of 4 × 4.5 m in Ethiopia and Tanzania, and of 3 × 3 m in Nigeria. Plants in the net plot were harvested and total fresh weights of cobs and stover were recorded. Out of the total cobs and stover harvested in the net plot, ten cobs and five stalks of stover were randomly selected and weighed. The grain and stover samples were oven-dried at 60 °C for the determination of dry matter weight. The five cobs were shelled and the shelling factor was calculated as the ratio of grain to total cob weight of the five cobs. The product of total cob weight (kg cobs/net plot) and the shelling factor (kg grain/kg cobs) is the maize grain yield (kg/net plot). Grain yield was then adjusted to 15% moisture content and converted to yield per hectare.

Grains and stover samples were taken, dried to constant weight and ground for determination of biomass nutrient concentration. The concentration of total nitrogen in the grain and stover was determined using a micro-Kjeldahl digestion method, while phosphorus and potassium were analysed based on Mehlich-3 extraction procedure preceding inductively coupled plasma optical emission spectroscopy.

### 2.3 Nutrient Expert (NE) performance trials

To evaluate agronomic and economic performance of fertilizer recommendations generated with Nutrient Expert, 368 trials were conducted in farmers' fields in the main crop growing seasons of 2016, 2017 and 2018. Maize yield responses to different nutrients, determined from the NOTs data, were used to guide the selection of these performance trial sites to cover a broad range of response domains in the major maize growing areas. A total of 58 and 108 field trials were conducted in Ethiopia and Tanzania, respectively. In Nigeria, two trials were conducted side by side in the same field to give a total of 202 field trials; one trial was for hybrid maize and the other one for OPV. In each study area in each country, the maize varieties used in the NOTs were also used in the performance trials.

The performance trials comprised of four treatments: (i) nutrient recommendations generated with NE, (ii) soil-test based nutrient recommendations (ST), (iii) the current blanket regional nutrient recommendations (RR) and (iv) a control plot (CR) with no nutrients applied. The attainable yields were determined from the NOTs and they were the same for each treatment in each study area.

#### 2.3.1 Generation of fertilizer recommendations with NE

NE was used to generate fertilizer recommendations at each experimental field. Once NE is calibrated and validated for a particular location with data from the NOTs, it estimates the attainable yield and yield responses to fertilizer from site information with decision rules developed from on-farm trials. To enable rapid collection of input data through the NE digital interface, data for a total of only five observable variables (minimum data input) are required to run NE software to generate reliable fertilizer recommendations. The attainable yield is estimated from two variables: (i) farmer's maize yield with current fertilizer management for a growing season with typical rainfall conditions, and (ii) characteristics of the growing environment: water availability (irrigated, fully rainfed, rainfed with supplemental irrigation) and any occurrence of flooding or drought. The growing environment is classified into: low-risk, medium-risk, and high-risk based on the probabilities of flooding or drought. The soil N, P and K supply classes for determining nutrient limited yield are estimated from three variables (i) soil characteristics (i.e. texture, color and content of organic matter), (ii) historical use of organic and inorganic inputs, and (iii) apparent nutrient balance (for P and K) from the previous crop (i.e. crop type, fertilizer input) (Pampolino et al., 2012). The input data are collected from host farmers and extension advisers through NE digital interface with simple questions.

#### 2.3.2 Development of fertilizer recommendations with soil-test based (ST) method

Before trial establishment, the soils were sampled with an auger from 0 to 20 cm depth from four points in each trial field using a V- random sampling scheme. The four collected samples from each field were thoroughly mixed to form a composite sample. The clods in the composite sample were crushed and the sample sieved through a 2 mm sieve for laboratory analysis. The soils were analysed for pH in water, soil/water ratio of 1:1, (measured with a glass electrode pH meter), texture (hydrometer method), total N (micro-Kjeldahl digestion), and available P and K were analysed based on Mehlich-3 extraction procedure preceding inductively coupled plasma optical emission spectroscopy. The secondary and micronutrients were not included in the analysis as the NOTs results demonstrated that overall maize yield responses to these nutrients were low across the studied sites in all three countries (see Table 3). Based on concepts described by Berger (1954), each of P and K input requirement was estimated for an attainable maize yield (Y_t_) with an eq. (8):

(8) $$N_{i}=\left(\mathrm{NR}\times Y_{a}\right)/\mathrm{RE}–\left(S_{i}\times E_{s}\right)/\mathrm{RE}$$

where N_*i*_ is the crop nutrient input requirement; *i* = P or K; NR is N, P or K requirement per tonne of maize grain; Y_a_ is attainable yield; S_*i*_ is soil available nutrients derived from chemical analysis; E_s_ is efficiency of soil nutrients, which is estimated from the ratio of a nutrient uptake (kg ha^−1^) to soil test value for available nutrient, derived from the nutrient omission plots.

**Table 3:** Maize yield response to fertilizer nutrients in major maize production areas in Nigeria, Ethiopia and Tanzania; the values are means, and the values in brackets are 25th and 75th percentiles.

Country	Study area	Yield response (t ha^−1^)				
		Nitrogen	Phosphorus	Potassium	Secondary- & micro-nutrients*	NPK
Nigeria	*States*					
	Kaduna (*N* = 72)	2.5(1.4–3.8)	1.8(0.8–3.0)	0.4(0.0–1.1)	0.3(0.0–1.4)	3.1(1.9–4.1)
	Kano (*N* = 48)	2.1(1.1–3.3)	1.5(0.2–2.5)	0.1(0.0–0.7)	0.3(0.0–1.1)	2.5(1.4–3.1)
	Katsina (*N* = 56)	2.6(1.3–3.9)	1.4(0.3–2.7)	0.0(0.0–0.7)	0.1(0.0–1.1)	2.6(1.4–3.7)
Ethiopia	*Regions*					
	Jimma/Bako (*N* = 80)	3.7(2.4–5.3)	1.0(0.2–1.8)	0.1(0.0–0.6)	0.0(0.0–1.0)	4.3(3.3–5.5)
	Adami Tullu/Bulbula (*N* = 36)	1.9(1.0–3.1)	1.4(0.8–2.3)	0.2(0.0–0.8)	0.3(0.0–0.9)	2.1(1.3–3.3)
	Hawassa (*N* = 30)	1.4(0.5–2.3)	0.4(0.0–1.3)	0.2(0.0–0.6)	0.0(0.0–0.4)	1.4(0.5–2.2)
Tanzania	*Zones*					
	Southern highlands (*N* = 108)	1.9(0.7–2.7)	1.2(0.3–1.8)	0.2(0.0–0.8)	0.2(0.0–0.8)	2.4(1.1–3.0)
	Northern (*N* = 66)	1.5(0.5–2.4)	0.3(0.0–1.0)	0.1(0.0–0.6)	0.1(0.0–0.6)	1.6(0.7–2.4)

The data for Nigeria and Ethiopia was averaged for two cropping seasons (i.e. 2015 and 2016). * Secondary- & micro- nutrients included calcium, magnesium, sulfur, boron and zinc. Yield response to N, P or K is the yield gap between NPK plots that received ample nutrients and omission plots when one of the nutrients is omitted; yield response to secondary nutrients & micronutrients was calculated as yield from plots supplied with NPK + secondary & micronutrients minus yield from plots supplied with NPK. Yield response to NPK was calculated as yield from plots supplied with NPK minus yield from plots with no fertilizer applied. The yield response data for OPV for Nigeria are not presented in this table because the responses were similar to this of hybrid.

The values of NR, S_*i*_, Ya, RE and E_s_ were derived from the NOTs data. The N input requirement for each experimental field was calculated from yield response agronomic fertilizer use efficiencies data generated from the NOTs, and confirmed from literature reported for SSA (Tittonell et al., 2008; Vanlauwe et al., 2010). The method we used for the soil-test based recommendations was an alternative after we recognized that there were no well-established recommendations developed based on this method in each country.

#### 2.3.3 Blanket fertilizer recommendations

The blanket regional recommendations (RR) were acquired from agricultural research institutions in each country. Blanket recommendations are the most commonly recommended type of fertilizer recommendations in Africa. They have been developed for large areas or agroecological zones, and are based on general soil and climate information using limited number of nutrient response trials, and usually take economic cost and benefits into consideration.

#### 2.3.4 Establishment and agronomic management of NE performance trials

The three different fertilizer recommendation treatments and the control were arranged in a randomized complete design and replicated across farms. The experimental fields were prepared with a plough using draught animals. The size of each experimental plot varied from 90 to 250 m^2^, depending on the size of the field offered by a farmer. Plant spacing of 75 × 25 cm was used to achieve a plant density of 53,000 plants ha^−1^. The nutrient sources were urea, single superphosphate (SSP) and muriate of potash (MOP) fertilizers. Nitrogen fertilizer was spot applied in three splits: at planting, at 21 and 42 days after emergence for recommendations generated with each method. All other nutrients were spot applied at recommended rates as basal at planting. Weeds, pests and diseases were controlled by following each country's recommended practice.

Maize grain yield was determined with the same method as used for the NOTs. The net benefit of each recommendation method (treatment) was estimated through a partial budgeting approach. Net benefit was calculated by subtracting the total costs that vary across the treatments (i.e. fertilizer cost) from the gross benefits for each treatment. The gross benefits for each treatment were calculated by multiplying the price of maize grain yield per kg by the maize grain yield (kg). Other costs of production such as labour for ploughing, planting, weeding and harvesting were not taken into account since they were similar for all four treatments. Given that the increase in costs is also important in determining the use of a technology by farmers, marginal rates of return were also calculated as the change in net benefits divided by the change in costs that vary, of alternative treatments, proceeding in steps from the least costly treatment to the most costly. This ratio is expressed as a percentage. The prices of fertilizer and maize grain yield at harvest were obtained from local agro-dealers and farmers.

### 2.4 Data analysis

Summary statistics including maximum, minimum, median, mean and standard deviation were used to explore the datasets of both the nutrient omission and performance trials for yield response to fertilizer nutrients, nutrient use efficiency. In accordance with the central limit theorem, the data were assumed to be normally distributed because their volume was large (*N* > 30). A generalized linear fixed model (GLFM) was used to test for significance of the effects of nutrient (in Genstat, version 9.2), season (in case of Nigeria and Ethiopia) and cultivar type (in case of Nigeria), and the two-way and three-way interactions on maize yield. All three factors (nutrient, cultivar and season) were included as fixed. The effects of fertilizer recommendations generated with Nutrient Expert, soil-test based and blanket recommendation methods on maize grain yield and nutrient use efficiencies were also analysed with the GLFM. Means were separated with Tukey's test at α = 5% level of significance.

## 3 Results

### 3.1 Maize grain yield response to applied nutrients

**Fig. 2:**
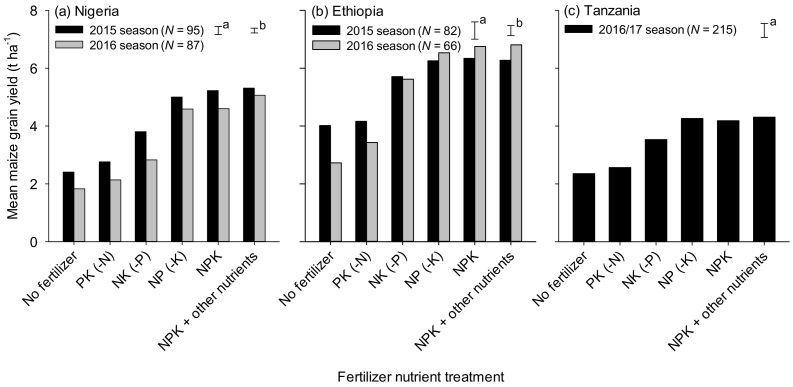
Maize grain yield for different fertilizer nutrient treatments in Nigeria and Ethiopia in two cropping seasons in 2015 and 2016, and in Tanzania in one cropping season in 2016/17. Yield data for OPV for Nigeria is not presented as they were similar to this of hybrid for each fertilizer nutrient treatment. Other nutrients refer to calcium, magnesium, sulfur, boron and zinc. Bars show the standard errors of difference of means for fertilizer nutrient treatment (a) and season (b). Error bars for all interactions are not shown as they were not significantly different.

**Fig. 3:**
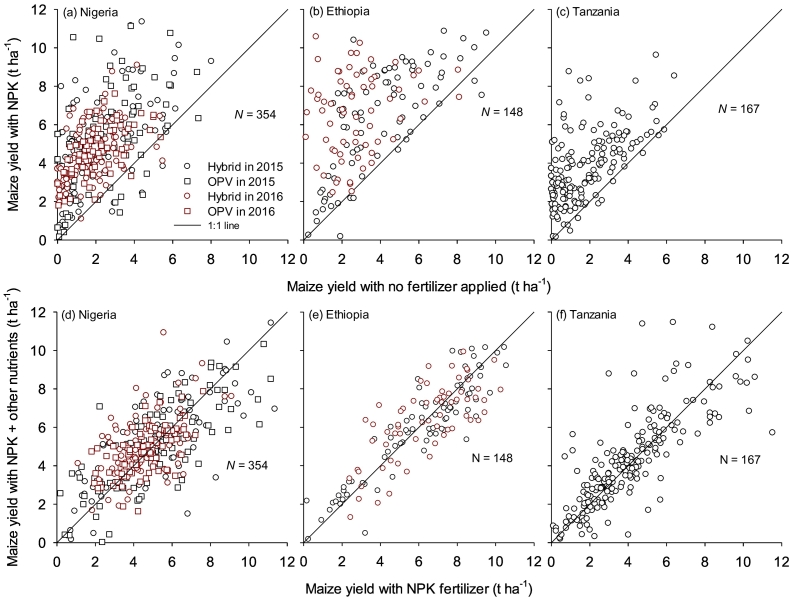
Maize grain yield response to soil amended with NPK fertilizers relative to maize grain yield for a control plot (no fertilizer applied) in Nigeria (a), Ethiopia (b), and Tanzania (c); and maize grain yield response to soil amended with NPK + other nutrients, relative to NPK fertilizers in Nigeria (d), Ethiopia (e) and Tanzania (f). Other nutrients refer to calcium, magnesium, sulfur, boron and zinc.

Maize grain yields were on average higher in Ethiopia than in Nigeria and Tanzania for each treatment, with maize yields for NPK treatment ranging between 3.8 and 7.5 t ha^−1^ in Ethiopia, 4.1 and 5.2 t ha^−1^ in Nigeria, and 3.7 and 4.6 t ha^−1^ in Tanzania (Fig. 2). In Nigeria, the maize grain yields realized from OPV and hybrid varieties were similar for each nutrient application category (data not shown), implying that similar nutrient management and fertilizer recommendations can be used for both maize varieties. Compared with the control (no fertilizer applied), maize yield increased significantly at most of the studied sites when NPK fertilizer was applied (Fig. 3a-c).

The maize grain yield response to nutrients supplied from fertilizer varied from farm to farm and from season to season. Maize yield response to N averaged between 2.1 and 2.6 t ha^−1^ in Nigeria, 1.4 and 3.7 t ha^−1^ in Ethiopia and 1.5 and 1.9 t ha^−1^ in Tanzania (Table 3). The response of maize yield to P was also positive in the majority of the studied locations, averaged between 1.4 and 1.8 t ha^−1^ in Nigeria, 1.0 and 1.4 t ha^−1^ in Ethiopia, and 0.3 and 1.2 t ha^−1^ in Tanzania (Table 3). Significant maize yield response to K, secondary nutrients and micronutrients were observed in specific areas in each country (Fig. 3d-f, Table 3). Maize yield increased by at least 1 t ha^−1^ when secondary and micronutrients were applied in Bunkure, Dandume and Soba LGAs in Kaduna, Kano and Katsina States of Nigeria, respectively. Secondary and micronutrients also increased maize yield in Adami Tullu in Ethiopia and Mbozi district in Tanzania.

### 3.2 Nutrient use efficiency

**Fig. 4:**
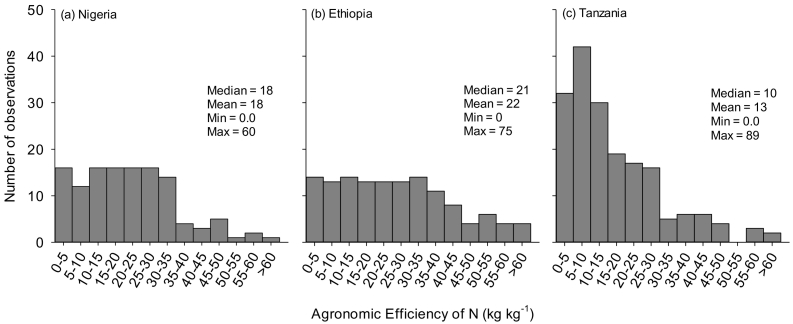
Agronomic efficiencies of nitrogen (N) for datasets generated from nutrient omission trials (NOTs) conducted in Nigeria, Ethiopia and Tanzania between 2015 and 2017.

The agronomic, recovery and internal efficiencies of N, P and K use varied with location. However, the most frequent scores of agronomic efficiencies (AE) of N are lower than 30 kg grain kg^−1^ N; on average 18, 22 and 13 kg kg^−1^ in Nigeria, Ethiopia and Tanzania, respectively (Fig. 4). The agronomic efficiencies of P and K were respectively 32 and 2 kg kg^−1^ in Nigeria, 21 and 3.6 kg kg^−1^ in Ethiopia and 15 and 0 kg kg^−1^ in Tanzania (Table 4). The recovery efficiencies of N, on average, ranged from 0.24 to 0.41 in Nigeria, 0.22 to 0.34 in Ethiopia, and 0.21 to 0.23 in Tanzania (Table 4). The recovery efficiencies of P were on average > 0.2 in Nigeria and Tanzania, and 0.1 in Ethiopia (Table 4). The recovery efficiencies of K were 0.5 in Nigeria, while it was 0.1 in both Ethiopia and Tanzania (Table 4).

**Table 4:** Recovery, internal nutrient (IE,) and agronomic use efficiencies calculated with data from nutrient omission trials (NOTs) conducted in Nigeria, Ethiopia and Tanzania between 2015 and 2017.

Country	Study area	Recovery efficiency			IE (maximum accumulation)			IE (maximum dilution)			Agronomic efficiency		
Nigeria	*State*	N	P	K	N	P	K	N	P	K	N	P	K
	Kaduna	0.41	0.3	0.56	42	273	53	84	601	111	17.5	34.5	5.4
	Kano	0.24	0.2	0.27	44	192	53	125	578	120	16.4	28.8	0.9
	Katsina	0.34	0.2	0.53	45	207	38	89	644	95	19.2	31.2	0
	Average	0.33	0.23	0.45	44	176	38	103	608	110	17.7	31.9	2.3
Ethiopia	*Region*												
	Jimma/Bako	0.34	0.08	0.04	–	–	–	–	–	–	29.9	20.8	2.3
	Adami Tullu/Bulbula	0.22	0.10	0.15	–	–	–	–	–	–	14	30.3	4.4
	Hawassa⁎	–	–	–	–	–	–	–	–	–	12.6	12.3	6
	Average	0.3	0.1	0.1	27	194	16	80	505	87	22.4	21.3	3.6
Tanzania	*Zone*												
	Southern highlands	0.21	0.31	0.17	35	100	28	88	604	129	14.7	24.1	0.5
	Northern	0.23	0.33	0.00	33	79	32	99	306	99	11	0.1	0
	Average	0.22	0.3	0.1	34	90	30	98	537	126	13.3	14.8	0

⁎ Nutrient content in maize above ground biomass was not analysed for Hawassa trials. The average IE for Ethiopia was derived from aggregated data from Jimma/Bako and Adami Tullu/Bulbula areas.

The average maximum accumulation and dilution of the internal nutrient use efficiencies were estimated at 44 and 103 kg grain kg^−1^ N, 176 and 608 kg grain kg^−1^ P, and 38 and 110 kg grain kg^−1^ K, respectively, in Nigeria (Table 4). In Ethiopia, the maximum accumulation and dilution of the internal nutrient use efficiencies were estimated at 27 and 80 kg grain kg^−1^ N, 194 and 505 kg grain kg^−1^ P, and 16 and 87 kg grain kg^−1^ K, respectively (Table 4). In Tanzania, the maximum accumulation and dilution of the internal nutrient use efficiencies were estimated at 34 and 98 kg grain kg^−1^ N, 90 and 537 kg grain kg^−1^ P, and 30 and 126 kg grain kg^−1^ K, respectively (Table 4).

### 3.3 NE performance relative to other fertilizer recommendation methods

**Fig. 5:**
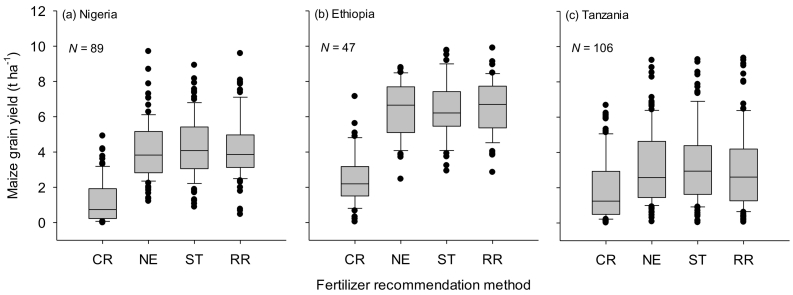
Maize grain yield from unfertilized control plot (CR), Nutrient Expert (NE), soil test based (ST) and blanket regional (RR) fertilizer recommendations in Nigeria, Ethiopia and Tanzania. The box and whisker plots show yield variations between experimental fields.

**Fig. 6:**
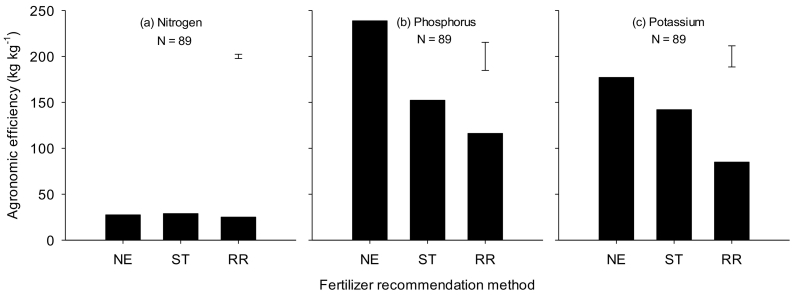
Agronomic efficiency of applied nitrogen, phosphorus and potassium for the Nutrient Expert (NE), Soil test based (ST) and Blanket regional (RR) fertilizer recommendations for the Nigeria study sites. Bars show the standard errors of difference of means for fertilizer recommendations generated with different methods.

**Fig. 7:**
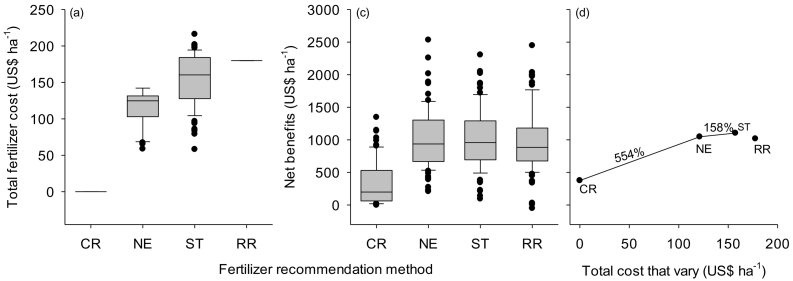
Cost of fertilizer (a) net benefits (b) and net benefit curve showing marginal rate of return (d) for control (CR) and fertilizer recommendations generated with Nutrient Expert (NE), Soil test based (ST) and Blanket regional (RR) in Nigeria. Exchange rate used: 1 US$ = 500 Naira. The box and whisker plots show variation in total fertilizer costs (a) and net benefits (b) between different experimental fields.

NE recommended lower amounts of phosphorus by 9 and 11 kg ha^−1^ than soil-test based and blanket regional fertilizer recommendations, respectively, in Nigeria (Table 5). Similarly NE recommended lower amounts of potassium by 24 and 38 kg ha^−1^ than soil-test based and blanket regional fertilizer recommendations, respectively, in Nigeria (Table 5). Yet, maize yields were not significantly different (at 4.4 t ha^−1^) for the three recommendation methods although the yields varied with location (Fig. 5a). Using less P and K nutrients while maintaining high yield levels, NE recommendations increased the agronomic efficiencies of P and K fertilizer use by 106% and 108%, respectively, over the blanket fertilizer recommendations (Fig. 6b & c). The mean agronomic efficiency of N was 27 kg kg^−1^ and was similar among the three recommendation methods in Nigeria (Fig. 6a). The cost of fertilizers used in maize production was lower by US$ 77 ha^−1^ for NE than for the regional fertilizer recommendations in Nigeria (Fig. 7a). Net benefits were also higher by US$ 30 ha^−1^ for NE than for blanket regional recommendations (Fig. 7b). On average, NE net returns are equal to 97% of the soil-test based net returns, while NE costs are 89% of soil-test based costs. NE is more profitable than soil-test based in almost half of all cases (46%). The marginal rates of return were higher for NE (554%) than for ST (158%) in Nigeria (Fig. 7c). Regional blanket recommendation was excluded from the net benefits curve because it has higher cost that vary, but lower net benefits.

**Table 5:** Median fertilizer rates for maize developed with three recommendation methods: Nutrient Expert, Soil-test based and Blanket regional, for Nigeria, Ethiopia and Tanzania in the 2016, 2017 and 2018 main cropping seasons. Values are median, and (minimum and maximum) in kg ha^−1^.

Country/nutrient	Fertilizer recommendation method		
	Nutrient Expert	Soil test based	Blanket regional
*Nigeria (N* *=* *101)*			
Nitrogen	110 (60–120)	123 (20–153)	120 (120−120)
Phosphorus	15 (4–23)	24 (0–35)	26 (26–26)
Potassium	12 (0–34)	36 (17–52)	50 (50–50)

*Ethiopia (N* *=* *58)*			
Nitrogen	120 (120−130)	120 (120–120)	111 (92–111)
Phosphorus	22 (17–32)	11 (0–29)	30 (30−30)
Potassium	26 (22–35)	0 (0–15)	0 (0–0)

*Tanzania (N* *=* *106)*			
Nitrogen	100 (100−110)	102 (81–118)	100 (100−100)
Phosphorus	12 (11–16)	22 (19–33)	9 (9–9)
Potassium	12 (11–14)	37 (26–48)	0 (0–0)

In Ethiopia, maize grain yield was not significantly different among the three recommendation methods (Fig. 5b) despite varied nutrient recommendations by the three methods (Table 5). NE and soil-test based methods respectively recommended lower amounts of phosphorus by 8 and 19 kg ha^−1^ than the blanket recommendations. However, the recommended N rate (120 kg ha^−1^) generated with NE was similar to soil-test based recommendations, but higher by 9 kg ha^−1^ than the blanket recommendations (Table 5). Fertilizer recommendations generated by all three methods did not require application of K fertilizer in Jimma and Bako regions of Ethiopia, as available soil K was adequate for maize production. However, NE recommended a median of 27 kg ha^−1^ of potassium (Table 5) for long-term soil fertility maintenance for sustainable crop production as NE is designed to take cognizance of the effect of nutrient mining on long term soil degradation. Consequently, in Ethiopia NE played a critical role as a co-learning tool between researchers and extension to further understand nutrient requirements in the two regions for improved recommendations.

In Tanzania, the median N rates generated with the three methods were similar although there were variations in NE and soil-test based recommendations (Table 5). The amount of P generated with NE and blanket recommendations were comparable, but they were respectively lower by 10 and 13 kg ha^−1^ than recommendations developed with the soil-test based method. The blanket fertilizer recommendations in Tanzania currently exclude recommendations for K fertilizers. However, NE recommended small amounts of K for soil fertility maintenance. Maize grain yields obtained between NE, soil-test based and regional fertilizer recommendations were not significantly different (Fig. 5c). However, maize yields obtained in Tanzania for all the three recommendation methods were highly depressed due to within-season dry spells and infestation by fall armyworm. The low maize yield affected the net benefits and hence they were low and comparable between the three methods (data not shown).

## 4 Discussion

The fertilizer recommendations generated with NE and soil-test based methods varied from farm to farm in each country. Consequently, the fertilizer rates developed from NE maintained high maize yield, and increased use efficiency of nutrients and net benefits of maize farmers. In particular, NE recommended lower amounts of P and K fertilizers, yet maize yields were similar to soil-test based and regional fertilizer recommendations in Nigeria. Using the available fertilizer blends in Nigeria, this meant a decrease in the use of NPK fertilizer, resulting in an investment saving of about US$ 80 ha^−1^. In Ethiopia, NE also recommended lower amounts of P than the blanket recommendations, but it maintained high yield levels. Using less P and K while maintaining high yields, NE recommendations thus increased the agronomic use efficiency of fertilizer. These results demonstrate that the blanket recommended P and K amounts in Nigeria and P amounts in Ethiopia, are higher than required. However, farmers normally apply lower rates of fertilizer than the recommended rates. At regional and global scales, excess P in the farming systems has a tendency of being washed away by rain water into water bodies leading to water pollution (Nyamangara et al., 2013).

In Ethiopia, NE and soil-test based methods recommended higher amounts of nitrogen than the regional recommendations. Yet similar yields were achieved with a lower rate of 111 kg N ha^−1^ from the blanket recommendation suggesting that NE and soil-test based recommendations underestimated the N that was supplied from the soil. Therefore, further work to improve the understanding of indigenous soil N supply in these regions in Ethiopia in relation to soil types and management history is needed to adjust the N rates recommended by NE. In this case NE was important as a co-learning tool among researchers.

All three recommendation methods were in agreement that in most of the studied sites, especially in Ethiopia, the soil K supply was adequate for maize production as no yield penalties were observed when K fertilizer was omitted. This is in accordance with the low maize yield responses to K observed in the nutrient omission trials (see Table 3), as also reported in literature (Nziguheba et al., 2009; Kihara et al., 2016). However, NE recommended small amounts of potassium for soil fertility maintenance based on SSNM principles that provide guidelines for maintenance K application in high potential maize production environments to avoid depletion of soil K reserves in the long-term (Dobermann and White, 1998). However, there is a trade-off between applying K for soil maintenance and profitability of a recommendation. The important implication of this is that there is need to revise the NE algorithm for applying K for maintenance of soil fertility to reduce K application for increased profits of maize farmers in Ethiopia.

The variability in fertilizer recommendations generated with NE and soil-test based methods is due to wide variability in yield responses to soil and fertilizer nutrients as influenced by varying inherent soil nutrient supply, attainable yield, recovery and internal nutrient use efficiencies (Tittonell et al., 2008). The soil nutrient supply, attainable yield and nutrient use efficiencies vary due to different soil types with varying mineralogy and soil water holding capacity, rainfall conditions, crop variety and crop agronomic management practices. Results from the nutrient omission trials conducted in this study demonstrated that maize yield responses to soil and fertilizer nutrients and maize yield vary with location and season.

Similar to yield responses, the agronomic and recovery efficiencies of applied nutrients also varied with study site. Although agronomic efficiencies of N (AEN) fertilizer vary with location, their mean values of <22 kg kg^−1^ observed in each country in this study were much lower than an average value of 36 kg kg^−1^ observed in well-managed farmers' fields with high organic carbon content (Kurwakumire et al., 2014) and when a combination of mineral fertilizers and manure or compost was used in maize in SSA (Vanlauwe et al., 2010). This demonstrates the importance of improved agronomic management practices to increase efficiency use of nutrients and productivity in these crop systems, especially in Tanzania where the values of AEN are too low (ten Berge et al., 2019). The low values of AEN were, however, comparable with the values reported for SSA for maize applied with mineral fertilizer alone (Smaling and Janssen, 1993; Vanlauwe et al., 2010).

The N recovery fractions of <0.4 observed in this study are lower than a standard value of 0.5 reported for maize under on-farm conditions in sub-Saharan Africa (Janssen et al., 1990; Smaling and Janssen, 1993). However, these low N recovery fractions are similar to values observed in poorly managed fields characterized by low organic carbon (Kurwakumire et al., 2014). The P recovery fractions observed in this study in Nigeria and Tanzania are slightly higher than a standard value of 0.1 reported for maize in SSA (Janssen et al., 1990). The higher values of P recovery fractions may be partly related to build-up of residual P in the soil due to continuous application of excess P fertilizers in the previous seasons. The K recovery fractions observed in this study in Ethiopia and Tanzania are lower than a standard value of 0.5 observed for maize in SSA (Janssen et al., 1990). The yield to nutrient uptake ratios of maximum dilution and accumulation of N, P and K observed in this study are within the ranges of values reported for SSA (Janssen et al., 1990; Smaling and Janssen, 1993; Tittonell et al., 2008) and other regions (Liu et al., 2006; Sattari et al., 2014) suggesting that these values are applicable to a wider range of maize growing conditions.

Overall, the wide variation in yield responses to soil and applied nutrients reinforces the need for development of decision support tools such as NE for site-specific fertilizer recommendations in SSA.

NE decision support tool has demonstrated its usefulness in formulating fertilizer recommendations that are specific to field conditions, but scaling its recommendations to new geographies demands establishment of fertilizer response trials or mobilization of legacy data from fertilizer trials. Due to considerable financial, time and infrastructural investments, it is difficult to continually establish multi-location on-farm diagnostic fertilizer response trials in a wide range of agro-ecologies. On the other hand, the legacy data on yield responses to nutrients is not available in many areas in sub-Saharan Africa. Geo-spatial data on soil, weather, crop yield, which have become increasingly available for public use, will create a great opportunity to understand crop response to soil and fertilizer nutrients for larger areas and enable rapid calibration of nutrient management decision support tools such as NE to be able to reach millions of farmers with nutrient management advice. For instance, the soil information project has improved the availability, quality and resolution of geo-referenced data on soil fertility properties and soil water, in SSA (Hengl et al., 2017; Leenaars et al., 2018). Similarly, HarvestChoice has increased the availability of spatial yield data (https://harvestchoice.org), while the Climate Hazards Group InfraRed Precipitation with Station data (CHIRPS) has increased the public availability of spatial weather data (http://chg.geog.ucsb.edu/data/chirps). However, groundtruthing of geospatial agronomic data is important to improve the predictions as well as to evaluate the uncertainties of fertilizer recommendations provided to farmers.

NE can be overlaid on emerging spatial frameworks as the technology extrapolation domain (TED), which is designed to scale out technologies and practices to larger areas (Andrade et al., 2019). The TED framework delineates an area with similar climate and soil factors (i.e. annual total growing degree-days, aridity index, annual temperature seasonality and plant-available water holding capacity in the rootable soil depth) and these factors govern crop yield response in rain-fed cropping systems. Consequently, NE can be used to provide a specific recommendation to a TED, as the domain is assumed to have a similar yield response to soil and fertilizer nutrients.

## 5 Conclusion

Over 700 nutrient omission trials conducted across a broad range of major maize producing areas in this study reinforced earlier observations that there is a wide spatial and temporal variability in crop yield response to soil and fertilizer nutrients in African smallholder cropping systems. Nitrogen was the most limiting for maize growth followed by phosphorus in each country in Nigeria, Ethiopia and Tanzania. Promoting fertilizers that balance soil N and P dynamics for balanced plant nutrition is important for increased yields and profits of small holder maize farmers as well as protecting the environment. The calculated QUEFTS envelope functions from this study were overall within the range of values reported for SSA and this demonstrates that QUEFTS is generic and can form a basis for developing simple nutrient management decision support systems such as NE that promote site-specific nutrient management. The nutrients recommendations generated with NE decision support system balanced fertilization and maximized the agronomic efficiency (AE) of applied nutrient inputs. It maintained high yields, but at a lower fertilizer input cost than current recommendation methods. Even though NE performed better, there is still a room to further improve its predictions as more knowledge about local maize production is acquired and capacity to improve the quality of input data is built. NE was effective as a simple and cost-effective tool for fine-tuning fertilizer recommendations to farm-specific soil fertility conditions in wide range of soil and climatic conditions in sub-Saharan Africa. However, NE recommendations need to be evaluated relative to farmers' current fertilizer practices as many farmers in Sub-Saharan Africa do not use recommendations generated with blanket or soil-test based methods. Geo-spatial soil and agronomic information that has become increasingly available for public use will create a great opportunity to enable rapid calibration of nutrient management decision support tools such as NE to provide recommendations for larger areas and reach millions of farmers with improved and sustainable nutrient management advice.
